# Depression, injecting drug use, and risky sexual behavior syndemic among women who inject drugs in Kenya: a cross-sectional survey

**DOI:** 10.1186/s12954-019-0307-5

**Published:** 2019-05-30

**Authors:** Catherine Mwangi, Simon Karanja, John Gachohi, Violet Wanjihia, Zipporah Ngang’a

**Affiliations:** 1 School of Public Health, Jomo Kenyatta University of Agriculture & Technology, Juja, Kenya; 2 Partners for Health and Development in Africa, Nairobi, Kenya; 3 Washington State University - Global Health, Nairobi, Kenya; 4 Centre for Public Health Research, Kenya Medical Research Institute, Nairobi, Kenya; 5 South Eastern Kenya University, Kwa Vonza, Kenya

**Keywords:** Syndemic, IDU, Depression, Intimate partner violence (IPV), Risky sexual behavior, Women who inject drugs (WWIDs), Kenya

## Abstract

**Background:**

Injecting drug use (IDU) is a key driver of the HIV epidemic particularly when individuals experience psychosocial conditions and risky sexual behavior in a syndemic manner. This study sets out to assess evidence of a syndemic pattern of psychosocial conditions (IDU, depression, intimate partner violence (IPV)) on one the hand and risky sexual behavior on the other while accounting for the socio-economic disadvantage among women who inject drugs (WWID) in low-income urban settings in Kenya.

**Methods:**

Using a cross-sectional study design, this study recruited 306 WWIDs from two sites in Nairobi between January 2017 and July 2017. Multiple methodologies including descriptive analyses of co-occurrences of psychosocial conditions at the individual level, standard logistic regression analyses to examine relationships and interactions within and between psychosocial conditions and risky sexual behavior, and classification trees algorithm for predictive modeling via machine learning were employed.

**Results:**

The prevalence of the psychosocial conditions was as follows: IDU, 88%; depression, 77.1%; and IPV, 84%. The prevalence of risky sexual behavior was 69.3%. IDU and depression were related to each other (*P* < 0.05) and each of them with risky sexual behavior (*P* < 0.05). The highest 2-way and 3-way co-occurrence of conditions were reported in IDU and depression (72%) and in IDU, depression, and risky sexual behavior (62%), respectively, indicating clustering of the conditions at the individual level. Further, each additional psychosocial condition (IDU and depression) was associated with sixfold odds (*P* = 0.000) of having risky sexual behavior suggesting a dose-response relationship. Logistic regression analyses incorporating multiplicative interactive effects returned three significant variables (*P* < 0.05): IDU*depression interaction effect, “Age when delivered the first child,” and “Income.” Classification tree modeling represented a 5-level interaction analysis with IDU and depression predicted to have the highest influence on risky sexual behavior.

**Conclusion:**

Findings provide possible evidence of a syndemic pattern involving IDU, depression, and risky sexual behavior suggesting the need for an integrated approach to the implementation of harm reduction interventions among WWID in low-income urban settings in Kenya. This work highlights the need for further studies to authenticate the findings and to characterize pathways in the syndemic development in WWID.

## Background

Given that injecting drug use (IDU) is a key driver of HIV epidemic through risky sexual behavior and use of contaminated injection tools [1], the majority of an estimated 3.8 million women and girls who inject drugs (WWIDs) globally are at risk of contracting HIV [2]. Among these WWIDs, certain psychosocial conditions including intimate partner violence (IPV) and depression co-occur within them leading to increased rates of risky sexual behavior and subsequent HIV transmission [3, 4]. For instance, HIV prevalence among People Who Inject Drugs (PWIDs) in sub-Saharan Africa is estimated to range from 5.5 to a high of 42.9% [5]. In Kenya, HIV prevalence among PWIDs is estimated at 18.7% [6] which is considerably higher than in the general population (5.6%) [7]. Broadly, the co-occurrence of IDU, depression, IPV, and risky sexual behavior has been reported across low socio-economic urban settings among WWIDs [4, 8].

Hypothesized relationships between psychosocial conditions and risky sexual behavior comprise a syndemic [9]. The syndemic approach is a novel attempt in global health research that challenges conventional frameworks that emphasize individual risk factor analyses in medicine and public health [10]. These conventional frameworks have been criticized as they often ignore social, economic, cultural, political, and ecological contexts [10]. Thus, the syndemic approach postulates that a common cause, such as low socio-economic disadvantage, underlies a syndemic [11].

The syndemic approach was originally developed by medical anthropologists to explain the population-level occurrence of HIV/AIDS in certain populations disproportionately affected by socio-economic disadvantage, social marginalization, gender-based violence, and other forms of social and environmental stress [12]. Previous research in diverse global regions has proven the existence of syndemics [10]. To name a few, Senn et al. outlined a syndemic pattern constituted of IPV, substance use, depression, and sexually transmitted diseases (STDs) among patients attending an urban STD clinic in the USA [13]. Distefano reported the presence of at least three syndemics in circumstances linking HIV with poor mental health, substance use, and violence in a synergistic manner in Japan [14]. Jiwatram-Negron et al. described a syndemic impact of injection drug use, IPV, and HIV on mental health among women using drugs in Kazakhstan [15]. The value of a syndemics approach in social epidemiology research is immense—the approach reveals clustering of exposures and outcomes of interest within populations and explains better the social, psychological, and biological factors on why and how exposures and outcomes of interest cluster within populations; the ways these factors interact with each other; the importance of these interactions to the health burden within the populations; and the pathways to the generation of these interactions [10].

A quick but thorough search of the scientific literature indicated that there are no studies that identified and characterized syndemics revolving around HIV burdens in sub-Saharan Africa including Kenya except in South Africa [16, 17]. Studies that have been conducted among PWIDs in Kenya have mainly been limited to the assessment of individual risk factors and behaviors associated with HIV incidence and prevalence missing out on the syndemic approach that advances a systems-thinking approach [18]. Further, these studies have employed small samples that lacked gender representativeness and gendered insights. For example, in one national integrated bio-behavioural survey conducted among PWIDs (*n* = 269), only 8.5% of the study participants represented women [6]. Similarly, in a rapid assessment survey of HIV and related risk factors among PWIDs (*n* = 344), WWID represented only 6.4% of the study participants [19].

To address the aforementioned limitations, this study reports the design and implementation of a large cross-sectional study in Kenya aimed at determining (1) the co-occurrence of IDU, IPV, depression, risky sexual behavior among WWIDs; (2) whether the interaction of psychosocial conditions (IDU, IPV, depression) was associated with higher rates of risky sexual behavior among WWIDs; and (3) whether the syndemic of IDU, IPV, depression, and risky sexual behavior was related to socio-demographic and socio-economic factors among WWIDs.

## Methods

### Study design and setting

This primary study adopted a cross-sectional study design. Study participants were WWIDs and were recruited from two sites supported by the Support for Addiction Prevention and Treatment in Africa (SAPTA) in Nairobi, Kenya. SAPTA is a non-governmental organization offering harm reduction services in low-income urban settings in Nairobi. WWIDs were enrolled from the SAPTA drop-in centers (DICs) in Pangani and Githurai study sites between January 2017 and July 2017 in Nairobi. The two study sites are urban-situated and in close proximity to each other within informal settlements. The study sites are also close to locations of injecting drug hotspots facilitating active PWIDs to approach program sites for services such as needle and syringe program among others. The two study sites offer WHO-recommended 9-element package [20] of PWID services and have a growing infrastructure and support for prevention, treatment, and care of PWID.

### Participant selection and recruitment

A total of 306 WWIDs participated in the study. To be included, participants had to be within the age bracket of 18–49 years to encompass the sexually active phase of interest in this study (risky sexual behavior), have a history of injecting drugs within one year preceding the study, and have had > 1 sexual partner consistently for 6 months preceding the study. The Kenya Medical Research Institute (KEMRI) Institutional Review Board approval was obtained before recruitment and participants were compensated with Ksh500 ($5). Targeted Mobilizer-Driven Sampling procedure that utilizes existing information about the study sites to systematically recruit study participants was employed in this study as outlined in Kral et al. [21]. Mobilizers who were peer educators recruited WWIDs from the injecting sites. The WWIDs who accepted the invitations based on availability were screened and scheduled for appointments.

### Data collection

The interviewer-administered questionnaire sought socio-demographics and socio-economic characteristics, IDU history, IPV, depression, and risky sexual behavior from the recruited WWIDs.

## Measures

### Demographics

Socio-demographic data was sought for age; highest level of education completed; average household income in the last 3 months; employment status; person responsible for income generation defined as self, spouse, combined self, and spouse and close relative; time in years lived in the informal settlement; reason for living in the informal settlement; marital status; religion; number of children; age at delivery of first child; and age when started living with a male partner.

### IDU

IDU was measured using the Diagnostic and Statistical Manual of Mental Disorders, Fifth Edition (DSM-V or DSM 5) criteria for substance use disorder [22]. WWIDs were asked if they experienced 11 different symptoms fitting into the DSM 5 classification as follows: impaired control over use, social impairment, risky use of the substance and pharmacological criteria.

To yield meaningful results, IDU was scored based on the extent of the problem with IDU dependent on the number of symptoms a participant identified fitting in the DSM 5 criteria for substance use (hereinafter referred to as IDU) disorder. The presence of 2 or 3 three symptoms indicated a mild IDU disorder; 4 or 5 symptoms indicated a moderate IDU disorder, and 6 or more symptoms indicated severe IDU disorder. These were further dichotomized as follows: mild was coded as 1 and moderate and severe was coded as 2.

### Intimate partner violence (IPV)

Physical, sexual, and psychological violence were measured using the revised conflict tactics scale (CTS2) [23]. The CTS2 scale measures the extent to which partners in dating, cohabiting, or marital relationship engage in physical, sexual, and psychological attacks or aggression. Its reliability ranges from 0.79 to 0.95. The conflict tactic scale includes subscales measuring the degree of severity of “less severe” and “more severe” behaviors. Women who reported having experienced any physical, sexual, or psychological acts of violence by her intimate partner with more severe scales in the preceding year to the study were recorded as having experienced IPV in the past year.

### Depression symptoms

The Center for Epidemiologic Studies Scale (CES-D) [24] was administered to assess depressive symptoms. This scale consists of 20 questions asking participants to report the frequency of depressive symptoms, that is, the number of days in the past week of experiencing depressive symptoms, for example, not being able to “shake off the blues,” having a hard time concentrating, etc. (CES-D) [24]. Responses to these questions were summed for a total score ranging from 0 to 60 points. Although this scale was used as a continuous measure, scores of 16 and above indicate a likelihood of clinical depression. This scale has been used in population-based and community studies [25]. The CES-D showed very good reliability (Cronbach’s *α* = 0.94). A score of 0–16 was coded as 1; a score of 17–60 was coded as 2.

### Risky sexual behavior for HIV infection

Risky sexual behavior was defined as a composite score based on the following variables: (1) number of casual male sexual partners in the last 6 months, (b) condom use during intercourse with casual male partners in the past 6 months, and (c) exchange of sex for either drugs or money to buy drugs with a casual male in the last 6 months. Casual partner was defined as “someone with whom the individual had sex one or more times without any regularity.” The following scores were attributed to the number of casual male sexual partners: none = 0, only one = 1, two to five = 2, six to ten = 3, and > 10 = 4. Similarly, scores for the number of times that a condom was used during intercourse with a casual partner in the past 6 months were the following: used a condom always with intercourse with a casual partner = 0, one to three times a month = 2, about once a week = 3, two to three times a week = 4, four to six times a week = 5, about once a day =6, two to three times each day = 7, did not use a condom during intercourse with casual partner = 8. Four dichotomous scores were created as follows: (1) did not have sex with a casual male partner in the 6 last month’s = 0 or had sex with a casual partner once or more times = 1, (2) used a condom always with a casual partner in the last 6 months = 0 or not use a condom always with a casual partner in the last six months = 1, (c) Exchanged sex for money to buy drugs with a male casual partner for the last 6 months, Yes = 1 or No = 0, and (d) exchanged sex for drugs with a male casual partner for the last 6 months, Yes = 1 and No =0. The four scores were summed up separately for each participant so that a participant could have up to 4 points. Those who declined to answer any of the four questions were excluded from the tally. The final overall score was calculated as the sum of the scores obtained for each individual participant where higher scores (≥ 2) indicated a greater degree of risky sexual behavior.

### Data analysis

Consistent with prior research, data was comprehensively analyzed in line with the three concepts that underlie the notion of a syndemic. We first assessed the extent at which psychosocial conditions co-occurred together in the study participants at the individual level. Using standard logistic regression models, we then estimated the magnitude of the relationship between the psychosocial conditions on the one hand and risky sexual behavior on the other. We subsequently assessed the additive effect of the psychosocial conditions on risky sexual behavior through building a separate logistic regression model with the count of psychosocial conditions (IDU, IPV, and depression) for each study participant as the independent variable and risky sexual behavior as the dependent variable. To further test for a syndemic, we assessed whether pairwise interactions of the psychosocial conditions predicted risky sexual behavior using additional logistic regression models adjusting for the underlying social and economic variables including age, time lived in informal settlement, reason for living in informal settlements, education level, religion, marital status, number of children, age at delivery of the first child, type of family the study participant grew in, income and its source, the one who works for the income, and age started living with partner.

Logistic regression analyses are global analyses characterized by a single predictive formula taking over the entire data space [26]. Building a single global model is difficult in instances of numerous variables interacting in complicated non-linear ways such as in this study. For this reason, we examined the data further by employing a recursive partitioning method that builds classification trees to better understand how the three psychosocial conditions and the host of underlying socio-economic variables interacted with each other to predict risky sexual behavior in WWIDs [27]. The classification tree was built via machine learning (ML) using a set of logical if-then conditions (instead of logistic equations in logistic regression) for predicting the outcome [27]. Specifically, this method involved splitting the data space (psychosocial conditions and socio-economic variables) into binary splits where the interaction analyses were more manageable. Each split was expressed by a series of if-then statements. As the ML algorithm took place, each variable descended the set of if-then conditions until a leaf of the tree was reached. If the if-then condition was true, the relevant category of a psychosocial condition or socio-economic variable went to the right branch of the tree; otherwise, it went to the left branch (27).

### Ethical considerations

This research was conducted in accordance with the World Medical Association’s provisions [28] governing research with human subjects. Data was collected in private rooms and restricted access to the information and coding of questionnaires was observed. Unique codes were used to track participants in lieu of names or other identifying information, and each participant signed a written consent and retained the right to withdraw participation anytime.

Ethical approval was provided by the Kenya Medical Research Institute (KEMRI), Ref KEMRI/SERU/CPHR/003/10/3242.

## Results

### Characteristics of the study participants

The mean age (standard deviation (SD)) of the study participants was approximately 30 years (5.7 years) whereas the median was 27 years. The highest proportion (32.7%) of the study participants were aged between 28 and 32 years (Table 1). The youngest and the oldest age of the study participants were 18 and 42 years, respectively.

**Table 1:** Characteristics of the sampled participants

	Characteristics	*N* (%)
Variable	Category	
Age	18–22	39 (12.7)
	23–27	79 (25.8)
	28–32	100 (32.7)
	33–37	59 (19.3)
	38–42	16 (5.2)
	> 42 years	13 (4.2)
Time living in informal settlement	1–10 years	65 (21.2)
	11–20 years	76 (24.8)
	21–30 years	96 (31.4)
	> 30 years	69 (22.6)
Reason for living in informal settlement	Place of birth	164 (53.6)
	Relocation	142 (46.1)
Education level	None	22 (7.2)
	Primary	184 (60.1)
	Post-primary	100 (32.7)
Religion	Roman Catholic	92 (30.1)
	Protestant	159 (52)
	Muslim	31 (10.1)
	No religion	24 (7.8)
Marital status	Married	21 (6.9)
	Cohabiting	277 (90.5)
	Single	8 (2.6)
Number of children	0	63 (20.6)
	1–3	123 (40.2)
	4–6	119 (38.9)
Age when got first child	11–15 years	67 (21.9)
	16–20 years	171 (55.9)
	> 20 years	68 (22.2)
Age commenced living with a partner	Under 18	165 (53.9)
	Over 18	141 (46.1)
Type of family grew in	Single parent	101 (33)
	Nuclear (father & mother)	157 (51.3)
	Extended (polygamous)	15 (4.9)
	Divorced/separated	33 (10.8)
Main means of living	Self-employed	44 (14.4)
	Theft	113 (36.9)
	Sex work	149 (48.7)
Income (every 3 months)	Kshs 0–10,000	120 (39.9)
	Kshs > 10,000	181 (60.1)
The one who works for income	Self	45 (14.7)
	Spouse	100 (32.7)
	Spouse & self	129 (42.2)
	Relatives	32 (10.5)

The mean time lived in the informal settlements was approximately 22 years (SD = 10 years) with a similar median time. The highest proportion of the sampled study participants (31.4%) had lived in the informal settlements for a period between 21 and 30 years. The time lived in the informal settlements ranged between 4 and 42 years, respectively. The study participants either lived in the informal settlement because they were primarily born there (54%) or due to relocation (46%) (Table 1). The mean and median number of children of the study participants was 3. Over half of the study participants (56%) got their first child between the age of 16 and 20 years. The mean (SD) and median age when the study participants got their first child was 18.5 years (3.2 years) and 18 years, respectively. Majority of the study participants (54%) commenced living with a partner when they were under 18 years and approximately 41% (*n* = 125) got their first child when they were under 18 years which is the recognized age of adulthood in Kenya. Fifty-one percent of the study participants grew up in a conventional nuclear family (Table 1).

The monthly mean income in Kenya shillings (Ksh) earned by the study participants within the last 3 months preceding the study was approximately Ksh12,877 (USD130). Majority of them (60%) obtained an income of more than Ksh10,000 (USD100). Financially, a combined 90% of the study participants depended on self, spouse, and both (self and spouse) (Table 1).

Other descriptive findings included the following: (1) majority of the study participants (60%) had attained primary level of education, (2) 80% were Christians, (3) almost all the study participants (90%) were cohabiting with intimate sexual partners while the rest were either married or single and (4) 49% reported that they employed sex work as their main means of living (Table 1).

### Occurrence and co-occurrence of psychosocial conditions and risky sexual behavior

Severe IDU was scored for 88% (*n* = 269) of the study participants, 77.1% (*n* = 236) met the threshold for depression, 84% (*n* = 256) had experienced IPV; while 69.3% (*n* = 208) met the threshold for risky sexual behavior.

The highest 2-way co-occurrence (72.2%) of psychosocial conditions among the study participants was reported in IDU and depression. The highest 3-way co-occurrence (62%) was reported in IDU and depression and risky sexual behavior (Table 2).

**Table 2:** Co-occurrence among psychosocial conditions and risky sexual behavior

2-way co-occurrence	Frequency
Depression + IDU	72.2% (*n* = 221)
Depression + IPV	66.0% (*n* = 202)
Depression + risky sexual behavior	62.7% (*n* = 192)
IDU + IPV	70.9% (*n* = 217)
IDU + risky sexual behavior	65.4% (*n* = 200)
IPV + risky sexual behavior	56.9% (*n* = 174)
3-way co-occurrence	
Depression + IDU + IPV	60.5% (*n* = 185)
Depression + IDU + risky sexual behavior	61.8% (*n* = 189)
IDU + IPV + risky sexual behavior	54.6% (*n* = 167)
Depression + IPV + risky sexual behavior	53.3% (*n* = 163)

### Associations among psychosocial conditions and risky sexual behavior

Study participants who met the threshold for depression were significantly (*p* < 0.05) more likely to report IDU and high sexual risk but not IPV (Table 3). Likewise, study participants who reported IDU were also more likely to report high-risk sexual behavior (Table 3).

**Table 3:** Unadjusted and adjusted odds ratios measuring the strength of associations between psychosocial conditions and risky sexual behavior

	Depression	IPV	IDU
IPV	OR = 1.8^a^ AOR = 1.7^a^		
IDU	OR = 4.0 AOR = 4.2	NC	
Sexual risk behavior	OR = 16.7 AOR = 17.5	OR = 1.1^a^ AOR = 1.0^a^	OR = 13.9 AOR = 16.6

^a^Not significant implying no association. *NC* model failed to converge

### Additive effect of psychosocial conditions and risky sexual behavior

A count of the number of psychosocial conditions (depression, IPV, and IDU) experienced by each study participant was associated with increased risky sexual behavior. There were sixfold odds of increased risky sexual behavior (95% confidence interval [CI], 3.8, 8.9) for each additional psychosocial condition experienced (Likelihood Ratio *χ*^2^ (degrees of freedom = 1) = 83.21, *P =* 0.000).

### Interaction effect of psychosocial conditions and social factors on risky sexual behavior

Logistic regression analyses that included risky sexual behavior as the dependent variable and pairwise interactions of each of the psychosocial conditions (depression, IPV, and IDU) and socio-demographic factors as independent variables returned only one significant interaction (Depression*IDU, *P* = 0.00) and two socio-demographic variables (Table 4). These variables were “Age when delivered the first child” and “Income” (Table 4).

**Table 4:** Logistic regression model predicting risky sexual behavior

Variable	Likelihood ratio test *χ*^2^ value	*P* value
Depression	0.06	0.81
IPV	1.67	0.20
IDU	1.41	0.23
Depression*IPV	0.89	0.35
Depression*IDU	15.19	0.00^€^
IPV*IDU	1.71	0.19
Age	1.35	0.24
Time lived in informal settlement	0.54	0.46
Reason for living informal settlement	0.36	0.55
Education	1.15	0.28
Religion	2.10	0.15
Marital status	1.13	0.29
Number of children	1.80	0.18
Age when delivered the first child	4.81	0.03^€^
Type of family grew up in	0.1	0.75
Source of income	1.92	0.17
Income	4.43	0.03^€^
The one who works for income	0.41	0.52
Age started living with partner	1.62	0.20

^€^Variable significant at *P* ≤ 0.05

*denotes an interaction term between the two variables

### Classification tree analysis for the risky sexual behavior

**Fig. 1:**
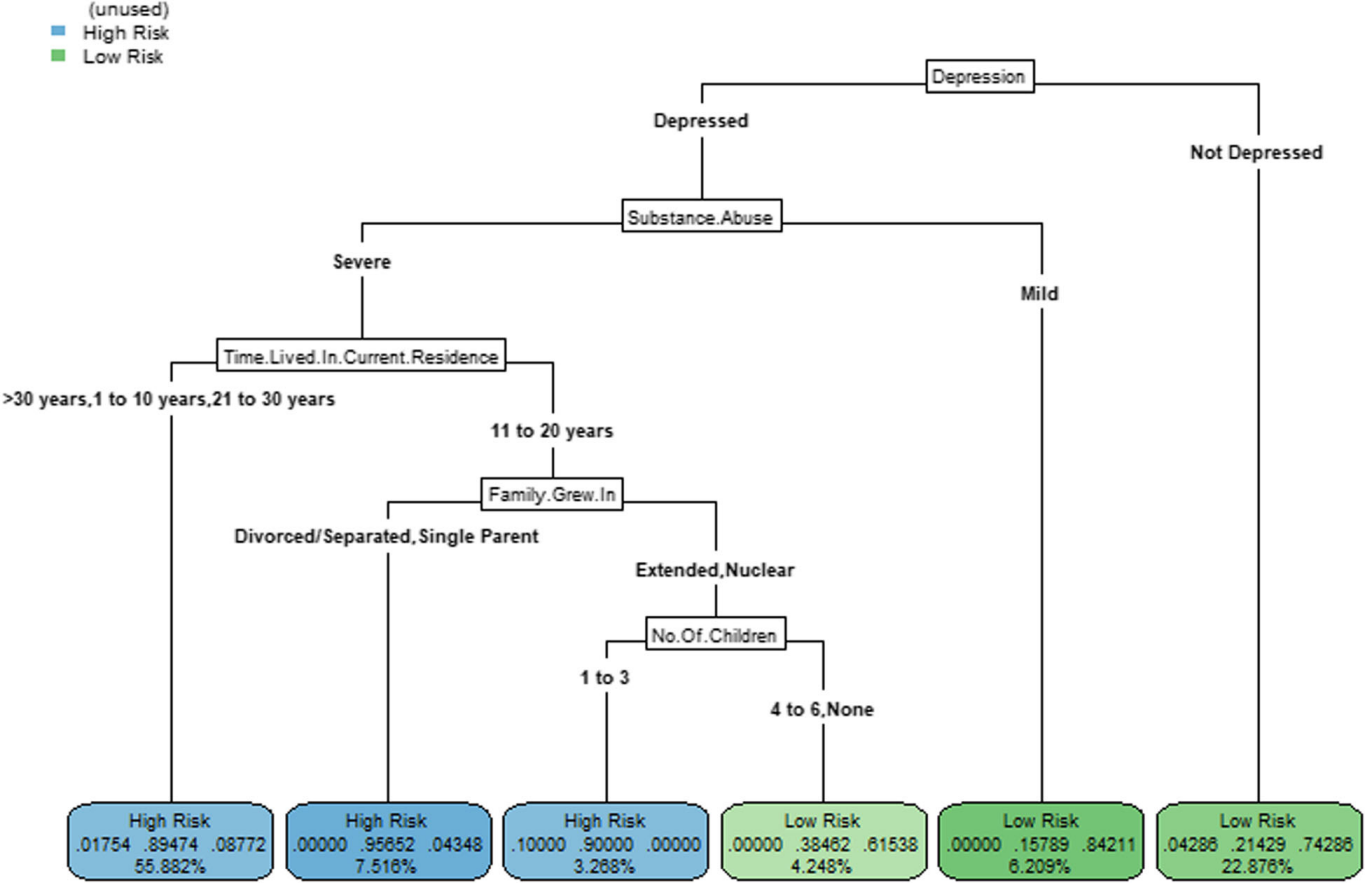
A classification tree of recursive partitioning of psychosocial, socio-economic, and socio-demographic variable space. High risk or low risk—those women participants identified with high or sexual risky behavior based on the number of casual male sexual partners in the last 6 months, condom use during intercourse with casual male partners in the past 6 months, exchange of sex for money to buy drugs with a male casual in the last 6 months, and exchange of sex for drugs with a male casual partner for the last 6 months. Depression: depressed—those women participants who had a score of 17–60 measure using the Center for Epidemiologic Studies Scale (CES-D) and not depressed—those women who had a score of 0–16 measure using the CES-D scale. Substance use: severe—those women participants with four or five symptoms measured using the Diagnostic and Statistical Manual of Mental Disorders, Fifth Edition (DSM-V or DSM 5) criteria for Substance use disorder, mild—those women participants with two or three symptoms measured using the DSM-V or DSM 5 criteria for substance use disorder. Time.Lived.In.Current.Residence—time in years lived in current residence stratified as 1–10 years, 11–20 years, 21–30 years, > 30 years. Family.Grew.In – type of family the women participants grew up in stratified as: single parent, nuclear family, divorced/separated and polygamous family. No.Of.Children – number of the children the woman participant has stratified as 0, 1-3, 4-6

The classification tree for risky sexual behavior is shown in Fig. 1. The resulting model (or the tree) considered five variables and, therefore, had five splits yielding six leaves. The tree represented a 5-level interaction because five variables were considered jointly to obtain the predicted value of risky sexual behavior (Fig. 1). Depression (*Depression*: Fig. 1) and injecting drug use (*Substance*.*Abuse*: Fig. 1) had the highest influence on risky sexual behavior (they were nearest to the root of the tree) (Fig. 1). Interactions involving variables time living in an informal settlement (*Time*.*Lived*.*In*.*Current*.*Residence*: Fig. 1), type of family they grew in (*Family*.*Grew*.*In*: Fig. 1), and the number of children (*No*.*Of*.*Children*: Fig. 1) were also influential in predicting risky sexual behavior.

The following partitions were classified as presenting high-risk sexual behavior: (1) those who met the cut-off for depression (*Depressed*: Fig. 1) with severe injection drug use (*Severe*: Fig. 1) and had lived in the informal settlements for either between 1 and 10 years (*1 to 10 years*: Fig. 1), 21–30 years (*21 to 30 years*: Fig. 1), or > 30 years (*>30 years*: Fig. 1) (55.9% of study participants); (2) those who met the cut-off for depression (*Depression*: Fig. 1) with severe injection drug use (*Severe*: Fig. 1) and had lived in the informal settlements for between 11 and 20 years (*11 to 20 years*: Fig. 1), grew up in divorced, separated, or single-parent families (*Divorced/Separated*, *Single Parent*: Fig. 1) (7.5% of study participants); and (3) those who met the cut-off for depression (*Depression*: Fig. 1) with severe injection drug use (*Severe*: Fig. 1) and had lived in the informal settlements for between 11 and 20 years (*11 to 20 years*: Fig. 1) and grew up in extended or nuclear families (*Extended*, *Nuclear*: Fig. 1), and with 1 to 3 children (*1 to 3*: Fig. 1) (3.3% of study participants). The following partitions were classified as presenting low risky sexual behavior: (1) those who did not meet the cut-off for depression only (*Mild*: Fig. 1) (22.9% of study participants), (2) those who met the cut-off for depression (*Depressed*: Fig. 1) but with mild injection use (*Mild*: Fig. 1) (6.2% of study participants), and (3) those with severe injection drug use (*Severe*: Fig. 1) and had lived in the informal settlements for between 11 and 20 years (*11 to 20 years*: Fig. 1), grew up in extended or nuclear families (*Extended*, *Nuclear*: Fig. 1), and with either no children or 4 to 6 children (*4 to 6*, *None*: Fig. 1) (4.2% of study participants).

## Discussion

Findings from this study demonstrate that IDU, IPV, and depression concentrate and interact to predict risky sexual behavior and occur under adverse social contexts among low-income urban WWIDs in Kenya. This pattern is consistent with prior research among low-income urban women in other countries [15, 29–31]. Besides, this pattern has been described and interpreted as reflecting a syndemic [11, 15, 32, 33].

A stringent interpretation of syndemic theory requires an empirical demonstration of three concepts: co-occurring psychosocial conditions in geographical contexts, interaction between the co-occurring psychosocial conditions that results in magnified adverse health and social consequences, and the influence of social contexts under which these psychosocial conditions occur. In seeking to meet these criteria, we applied multiple methodologies whose outcomes concurred in finding the possible presence of a syndemic. While previous studies have documented findings similar to our study [34–36], this is the first study to identify and quantify a syndemic among a sample of low-income WWIDs in Kenya.

This study found a high prevalence of psychosocial conditions at the individual level with approximately more than two thirds of the studied WWIDS having either of each of the psychosocial conditions (IDU, IPV, depression). Furthermore, at the individual level, more than half of them had a 2-way combination of IDU and/or IPV and/or depression. More harmful still, at the individual level, more than half of them had a 3-way combination of these conditions. Each of these conditions, alone or in combination, co-occurred with risky sexual behavior. Previous studies have reported that risky sexual behavior mediates the relationship between psychosocial conditions and HIV transmission [37–42]. Consequently, our findings suggest a clustered risk for HIV transmission among this population and fulfilled the first core feature of the syndemic concept. These co-occurrences could be bi-directional without a concrete understanding of what comes first, for instance, depression and the risk of undergoing and committing IPV may be aggravated by drug use and vice versa [43]. One of the key reasons of co-occurrence at both individual and population levels is their insidious onset accompanied with inadequate recognition and delayed attention suggesting a need for programming and longitudinal research that addresses this co-occurrence.

It is noteworthy that depression and risky sexual behavior considerably co-occurred in WWIDs in this study. Depression, which was not a component in the original syndemic concept consisting of substance abuse, violence, and AIDs (12) since its elaboration > 20 years ago, is emerging in recent research as an important element in syndemic involving increased HIV risk [15, 30, 41, 44, 45]. Previous studies suggest a higher frequency of depressive symptoms among PWID compared with the general population [45]. Further, bi-directional relationships between depression and risky sexual behavior have been reported with risky sexual behavior as a risk factor for depression [46] and depression escalating vulnerability to risky sexual behavior [47]. Whichever direction is taken, depression may harm brain-based skills needed for memory and to carry out tasks, lead to uncharacteristic social and or physical behaviors that may be harmful to others with adverse social consequences, contribute to psychosocial harm, reduced motivation, and unhealthy peer relationships [47, 48]. An external pathway to depression has also been hypothesized. This involves progressive criminalization of IDU and sex work among women that may lead to compounded stigma resulting in depressive symptoms and poor health seeking [42]. Our study did not have the capacity to measure these psychological and physical sequels of depression but nevertheless remains an area of promising research to establish cause-effect relationships.

Further analyses using logistic regression in this study found that depression and IDU interacted multiplicatively to increase the likelihood of risky sexual behavior among WWIDs in our settings. This analytical approach has been adopted in testing of syndemic [13]. Our logistic regression findings are consistent with previous studies that WWIDs experiencing depression engaged in unprotected sex, transactional sex for money or drugs, sexual relationships with partners who inject drugs, and disproportionately higher number of sex partners in their life course [35, 38, 41, 44]. Incidentally, these were the parameters we adopted in our study in defining risky sexual behavior among WWIDs.

Generally, an additional aspect of the interaction of these psychosocial conditions under the syndemic concept is that the conditions should manifest dose-response relationships such that a higher risky sexual behavior is reported among study participants who report a greater number of psychosocial conditions [13]. In our study, each additional psychosocial condition (injection drug use and depression) experienced by WWIDs was associated with approximately sixfold odds of increased risky sexual behavior which was clearly consistent with the conventional dose-response relationship [11, 15]. Consistent with previous research, the simultaneous presence of depression and injection drug use is elevated in women with sexual risk-taking histories by impairing judgment [25, 45]. Additional research suggests that injection drug use may serve as a form of self-medication for depression [49]. These findings on the interaction of psychosocial conditions to predict risky sexual behavior fulfilled the second criterion of a syndemic from multiplicative (logistic regression) and additive (dose-response relationship analyses) data analytic approaches.

Consistent with the third criterion in syndemic theory [10], this study found that the syndemic was associated with social-economic disadvantage variables of income and age at delivery of the first child. Occurrence of psychosocial conditions alone may not always lead to adverse health outcomes. Rather, consistent with previous research, conditions associated with living in low-income settings, such as overcrowding, underemployment, financial, and other stresses, and exposure to violence exacerbate at both individual- and population-levels to influence early sexual risk taking [10]. By identifying social variables of income and age at delivery of the first child, this study underscored the importance of a life course perspective that considers critical periods, in this case, teen pregnancies, and household economies for better understanding of processes, pathways, and stages of syndemic development.

We were expecting to identify IPV as a significant psychosocial condition in the syndemic as reported in other studies [3]. Indeed, IPV alone has been reported to escalate the risk for HIV transmission in women, including those engaging in IDU [50]. Further studies are needed to characterize IPV in this population given our finding of its co-occurrence with other psychosocial conditions but fell out in regression and interaction analyses.

By wanting to generate empirical support for the theory of syndemic, this study operationalized the concept of syndemic interaction pattern using global (logistic regression) and classification tree models. The overarching justification for applying multiple methodologies was not only to fill the gaps in knowledge existing in WWIDs interventions but also provide an evidence-based need for inclusion of joint interventions that address co-occurring and interacting conditions, hitherto known or unknown, that can lower HIV-related risky sexual behavior. To broadly test for the syndemic effect, we introduced an interaction term into the logistic regression and in addition used classification trees (models that employ recursive binary splits to relate an outcome and predictor variables). Both methodologies concurred in identifying the interaction between depression and injection drug use in predicting risky sexual behavior and demonstrated the practicality of considering a comprehensive syndemic framework. The implication of the latter finding is that an intervention addressing depression would be predicted to have a greater preventive impact if integrated with an intervention addressing IDU than would otherwise be predicted by analyses without the interaction term. These findings are an important addition to the existing policies that focus on an integrated prevention approach in HIV prevention among key populations in Kenya where routine screening of depression among PWIDs is sub-optimal.

Routine screening of depression and other mental disorders among people who inject drugs (PWID) is substantially limited in Kenya due to a shortage of human and other resources to effectively care for these disorders, stigma related to mental illness and treatment infrastructure issues, and limited awareness of the importance of mental health issues among others implying that early identification and treatment of mental disorders is challenging. Findings from this study, therefore, highlight the urgent need of recognizing depression as a major component of a syndemic among WWID in Kenya. For instance, integrating depression screening in programs such as the medically assisted therapy (MAT) and developing strategies to intervene effectively with WWIDs may reduce risky sexual behavior and improve overall population health outcomes.

Despite the strengths manifested in the approach of data collection and analyses in this study, our work had several limitations. To start with, the cross-sectional study design cannot allow confirmation of causality. A cross-sectional study design may provide varied findings if the study had been carried out in another timeframe. Additionally, the self-reported data in this study may have been limiting because some participants may have felt uncomfortable disclosing information pertaining to high-risk sexual behaviors, IPV, and depressive symptoms which are highly stigmatized especially among women. For that reason, information on the magnitude of harm associated with these conditions is unknown.

Notwithstanding these limitations, our findings indicate the urgent need to integrate the diagnosis and management of depression and other mental disorders as well as approaches focused on improving sustainable livelihoods among WWID in a joint approach to break barriers to the realization of zero new HIV infections by 2030. Even as we recognize that a high level of stigma and discrimination limits the use of psychiatric services in specialized facilities for psychiatric treatment in Kenya, interventions to increase referral and uptake from/to mental treatment are needed among PWIDs. Specifically, lessons learned from other programs, for instance, integration of psychiatric disorders treatment in the methadone maintenance treatment which has proved to be a successful model for PWIDs may be a useful starting point.

## Conclusion

Through diverse analytical methodologies, this study provided evidence of co-occurrence of IDU, IPV, depression, and risky sexual behavior among WWIDs in Kenya. Further analyses demonstrated interactions among IDU and depression to result in escalated risky sexual behavior under the contexts of socio-economic disadvantage. These three core features (co-occurrence, interactions, and context) define a syndemic concept that offers a novel way of understanding why increased risky sexual behavior clusters in populations experiencing harmful psychosocial conditions. These findings underscore the need for an integrated approach to the implementation of harm reduction interventions comprised of mental health screening and design of human-centered economic opportunities among WWID in low-income urban settings in Kenya. This work also highlights the need for further studies to authenticate these findings and to characterize pathways in the syndemic development in WWID.

## Data Availability

Due to the nature of the study and criminalized nature of drug use in the study context, entire data and materials from this study are not publicly available. Requests for data can be submitted to the corresponding author.

## Abbreviations

FGD: Focus Group Discussion.
IPV: Intimate Partner Violence
ML: Machine Learning
NSP: needles and syringes Programs
PWID: people who inject drugs
PWID: People Who Use Drugs
SA: Substance Use
WWID: Women Who Inject Drugs
